# Automating the Production of Communicative Gestures in Embodied Characters

**DOI:** 10.3389/fpsyg.2018.01144

**Published:** 2018-07-09

**Authors:** Brian Ravenet, Catherine Pelachaud, Chloé Clavel, Stacy Marsella

**Affiliations:** ^1^Laboratoire Traitement et Communication de l'Information (LTCI), Télécom ParisTech, Paris, France; ^2^Centre National de la Recherche Scientifique, Institut des Systèmes Intelligents et Robotiques, Sorbonne University, Paris, France; ^3^College of Computer and Information Science (CCIS), Northeastern University, Boston, MA, United States

**Keywords:** metaphorical gestures, embodied conversational agents, communicative behaviors, text analysis, embodied cognition

## Abstract

In this paper we highlight the different challenges in modeling communicative gestures for Embodied Conversational Agents (ECAs). We describe models whose aim is to capture and understand the specific characteristics of communicative gestures in order to envision how an automatic communicative gesture production mechanism could be built. The work is inspired by research on how human gesture characteristics (e.g., shape of the hand, movement, orientation and timing with respect to the speech) convey meaning. We present approaches to computing where to place a gesture, which shape the gesture takes and how gesture shapes evolve through time. We focus on a particular model based on theoretical frameworks on metaphors and embodied cognition that argue that people can represent, reason about and convey abstract concepts using physical representations and processes, which can be conveyed through physical gestures.

## 1. Introduction

Today, computers are essential in a wide range of activities, from solving mathematical problems to mediating our social interactions. Leveraging growth in computational power and functionality, researchers in the field of Embodied Conversational Agents (ECAs) aim to develop computer systems that can engage users in natural interactions. ECAs are virtual characters, usually with human-like appearances, endowed with the ability to use natural language and nonverbal behaviors the same way humans would (Cassell, 2000). They can be used as pedagogical assistants (Harvey et al., 2015), video-game characters (Gris et al., 2016) or they can also be integrated in more complex social simulations for medical purposes (Lisetti et al., 2015). Because their effectiveness relies on the user interacting with them the same way she would with another human, ECAs need to be able to decode and reproduce complex human communicative signals. While using only verbal communication may be satisfying for inputting basic commands, face-to-face communication requires the combination of speech with nonverbal behaviors that allows other communicative functions to be expressed simultaneously. For instance, research has highlighted how nonverbal behaviors are used by humans for disambiguation, clarification (Calbris, 2011), turn-taking management (Duncan, 1972) and socio-emotional expression while talking (Argyle, 1972). Therefore, in order to develop richer and more efficient natural interactions, ECAs require not only verbal capabilities but nonverbal ones as well.

While many communicative functions and nonverbal behaviors could be addressed, in this paper we focus on representational gestures which are gestures used to accompany and illustrate the content of the speech. In particular, we present an approach to automatically producing metaphoric gestures that are aligned with the speech of the agent in terms of timing and meaning (Cienki and Müller, 2008).

Metaphoric gestures use the physical behavior of a gesture, its form and motion, to convey abstract concepts. For example, although ideas are immaterial, a gesture that is a sideways flip of the hand can convey the speaker's rejection of an idea, as if an idea is a physical object with physical features such as form and location and therefore it can be held and discarded. This view is in line with the embodied cognition theories that argue that the same set of sensory and motor representations we use to make sense of and act in our world are also used to make sense of, reason and communicate about abstract concepts (Barsalou, 1999; Kendon, 2000; Tversky and Hard, 2009). Thought, and the message to convey, is therefore construed in terms of concrete elements, the properties of those elements and actions on them. In this way, an “idea” conceptualized as a concrete object possesses physical properties, such as size, location or weight, that are tied to the abstract properties. For example, an important idea is an idea that is big in size, ideas can be thrown away, etc. Beyond offering a physical representation to abstract elements, embodied cognition considers that reasoning and thought processing are actions taken on these representations (Johnson-Laird, 2006), and that gestures, in particular metaphoric gestures, are physical representations of these actions realized at the conceptual level (Hostetter and Alibali, 2008, 2010). In other words, holding an idea in our hand or rejecting it by a sideway flip of the hand is a mirroring of actions taken at the conceptual level, in effect, considering an idea to examine or dismiss it.

This work explores theoretical frameworks on metaphors and on how people represent and transfer physical properties from one concept to another that have been highlighted by researchers in the field of embodied cognition (Wilson and Golonka, 2013). We aim at capturing and understanding the specific characteristics of communicative gestures in order to envision how an automatic communicative gesture production mechanism, inspired by these theoretical foundations on human embodied cognition and on related work, could be built. Gesture characteristics (e.g., shape of the hand, movement, orientation or timing with respect to the speech) should convey the desired meaning. A system capable of producing automatically relevant and meaningful gestures is of particular interest for ECAs as they often rely on canned templates or on scripted scenarios. Due to the growing popularity of procedurally generated content in virtual worlds, a system that can control autonomously the verbal and the nonverbal behaviors of virtual characters could be used in a variety of applications, from video games and movie tools to virtual assistants. Our work faces the following challenges: identifying a common representation between speech and gestures that could be computationally manipulated, proposing a mechanism to extract semantic elements of this representation from the speech of the agent, associating these elements to gesture characteristics and finally combining these gesture characteristics to align them with the speech of the agent. Throughout this article, we detail the different conceptual components of our architecture and also their preliminary implementations to demonstrate the feasibility of such a system. While we aim for a balanced description of each of the conceptual components, some of them are more advanced in terms of implementation and will have a higher level of technical detail.

This article is organized as follows. In section 2, we present the theoretical foundations of our study on gestures, embodied cognition and discourse. We accompany this review with a discussion on the challenges that arise from replicating these human phenomena within an ECA. In section 3, we review and analyze existing solutions that tackled parts of our challenge. We leverage this literature to propose a system capable of generating metaphoric gestures starting from the text to be said by the virtual agent (see section 4). Finally, in section 5, we discuss the limits and perspectives of our approach and outline the requirements for future evaluations.

## 2. Gestures and meanings

While talking, humans produce various nonverbal behaviors that accompany the discourse. Among these behaviors, communicative gestures can carry different meanings. They can illustrate an idea, mimic an action or the shape of an object, indicate a point in space or even mark an emphasis (McNeill, 1992). Various taxonomies of gestures have been proposed to encompass these varieties of meaning (McNeill, 1992; Kendon, 2004; Poggi, 2007). Gestures can also be studied according to their functions in the communication process. For example, they can have a demarcative function and mark the rhythm of an utterance, so as to underline speech chunks or to coordinate who has the speaking turn. They can also be tightly tied to dialog acts underlying a speaker's intention. But gestures can also reveal a speaker's attitude toward what she is saying such as her level of certainty or of agreement. Additionally, gestures can carry information about affective states (Bänziger et al., 2012).

To convey these varieties of functions, the form and timing of gesture production in relation to speech is critical. The temporal relationship between speech and gesture is far from being trivial as gesture can coincide with speech prosody or can be anticipated or maintained afterward (Kendon, 2004). Additionally, gesture shape and movement carry important meaning.

In Wagner et al. (2014), the authors gave an extensive review of work on communicative gestures, from psychology studies to computer systems. The results highlighted how closely tied together speech and gesture are (in terms of meaning and timing). According to some theoretical models, like McNeill's *Growth Point Theory* (McNeill, 1985), this could be explained by the fact that gestures and speech are produced from the same mental process. In particular, many studies investigated the effect of embodied cognition on speech and gesture production (Hostetter and Alibali, 2008) and hypothesized the existence of a common mental imagery between the two communicative channels (Kendon, 1980).

### 2.1. Gestures—types and structures

Some scholars have underlined how gesture definitions, in term of shape and movement, can be viewed as the abstraction of an action (Kendon, 1980; Calbris, 2011). This is particularly true for metaphoric gestures. For example, rejecting an idea can be conveyed by a hand gesture metaphorically mimicking rejection with a pushing away gesture.

Gesture can be characterized by its physical constituents. The form of a gesture is described in term of the shape of the hand, the wrist and the palm orientation. A gesture can be made with one or two hands, symmetrically or in opposition. The movement of a gesture can be defined by its direction, its path, its dynamism.

As mentioned by Kendon (1980), gestures exhibit different structures. At the level of a gesture, there are different phases (e.g., preparation, stroke, hold and relaxation). Consecutive gestures can be co-articulated, meaning that the last phase of a gesture is mapped to the beginning phase of the next gesture. There is a higher structure that corresponds to discourse segments in which consecutive gestures share some of their constituents and are kinetically segmented. It corresponds to the ideational structure introduced by Calbris (2011). In her theory, Calbris argues that discourse is composed of units of meaning and rhythm she calls *Ideational Units*. Within an Ideational Unit, there is a consistency between the gestures of the person as they show similar properties.

### 2.2. Conceptual metaphors and image schemas

Within the literature on embodied cognition, the *conceptualization hypothesis* states that the way we mentally represent our world is constrained by our body (Wilson and Golonka, 2013). In other words, our interactions with the world through embodiment lead to the conceptual representations we manipulate in our mind to ground abstract and concrete concepts. This is how we can apply physical properties to abstract concepts as part of our metaphorical reasoning. Lakoff and Johnson (1980) describe *Conceptual Metaphors* to explain how we can talk about one domain using properties from another one. For instance, in the conceptual metaphor LOVE IS A JOURNEY, love is seen as having an origin, a destination (might be an end) and a series of events or steps between the two.

In that case, how do we represent in our mind these properties that can be shared between concrete and abstract entities? Johnson suggested that humans use recurring patterns of reasoning, called *Image Schemas*, to map these conceptual metaphors from an entity to another (Johnson, 1987). These *Image Schemas* have also been studied by Grady in order to attempt to explain how our perception mechanisms are at the origin of our metaphorical reasoning (Grady, 2005).

For instance, the *Image Schema* CONTAINER gives an entity the typical properties of a container such as having a boundary with elements that are within it and elements that are outside. We can think of culture metaphorically as a container in terms of people that are part of the culture, and people that are not. This illustrates how people use their physical reality to reason about abstract concepts, thus giving physical attributes to abstract concepts. Moreover, according to Wilson, using metaphoric reasoning can unconsciously influence our nonverbal behavior: if someone is thinking about a future event, he might be swaying slightly forward (Wilson and Golonka, 2013).

### 2.3. Image schemas and gestures

While these *Image Schemas* have been investigated as linguistic structures (Croft and Cruse, 2004), used in the production of speech, other work suggests that they could be at the origin of the accompanying gesture production as well (Cienki, 2013). In Mittelberg (2008), the author describes how a gesture (mimicking the shape of a box) can represent the *Image Schema* OBJECT or CONTAINER, itself being linked to the conceptual metaphor IDEAS are OBJECTS. In other work, Cienki conducted an experiment to study if *Image Schemas* (a subset) could be used to characterize gestures (Cienki, 2005); his conclusions showed positive results. In Chui (2011), the authors revealed evidence of the use of spatial conceptual metaphors in gesture production for mandarin speakers. Another experiment by Lücking and his colleagues tried to find recurrent gestures features in the expression of particular *Image Schemas* (Lücking et al., 2016). Their results showed that, for some *Image Schemas*, people spontaneously used similar gesture features. Finally, in Mehler et al. (2015), the authors developed a gesture-based interface for an interactive museum system that used *Image Schemas* as a basis for their gestural grammar.

Metaphorical reasoning allows the transfer of properties from a source domain to a target domain and, in the discourse, this is realized by talking about the target domain as if it was an entity of the source domain (Lakoff and Johnson, 1980). Metaphoric gestures follow a similar process and, like iconic gestures, their characteristics serve to illustrate and demonstrate particular physical properties (metaphorically projected in the case of metaphoric gestures) of the concept being communicated by the speaker (Cienki, 1998). A hypothesis is that these characteristics are tied to the *Image Schemas* underlying the production of the metaphorical reasoning. Researchers have highlighted how some typical metaphorical properties are often represented with the same gesture characteristics (Cienki, 1998; McNeill, 1992; Calbris, 2011). For instance, to represent the CONTAINER concept, one might exhibit concave hands facing each other in a bowl-like shape. These findings are in line with earlier works of McNeill and Levy who observed how people (through the use of the conduit metaphor Reddy, 1979) illustrate an abstract entity, which could be tied to the OBJECT *Image Schema*, by pretending to hold an object with their hand (McNeill and Levy, 1980).

More examples are given in these works. They illustrate that different characteristics are used depending on the metaphorical element being portrayed. Whereas CONTAINER and OBJECT seem to be depicted through the shape and the orientation of the hand, other *Image Schemas* can be portrayed by other physical characteristics such as the position or the quality of the movement. The *Image Schema* SPLIT, which would underlie a separation or a difference, can be illustrated by a vertically flat hand moving abruptly downward; the SCALE *Image Schema*, parameterized so it encapsulates the action of an increasing scale, can be depicted with both hands moving away from each other (Calbris, 2011).

Inspired by this research, we propose to use *Image Schemas* as the basis for our representation, to bridge the speech of an ECA and its gestures.

### 2.4. Gestures and speech alignment

Timing is key to the alignment of speech and gestures. For example, in McNeill's Growth Point Theory McNeill (1985), the growth point is the initial form (or seed) of the thinking process from which the future speech and gesture are constructed together in synchrony with each other. While *Image Schemas* are good candidates for predicting gesture shapes, additional information will be required in order to identify the most appropriate meaning to be aligned with the speech by the gesture production (not all *Image Schemas* are turned into gesture; selection happens). Even if each word in isolation carried an embodied meaning represented by an *Image Schema*, people do not produce a gesture on every word. For instance, an “important obstacle” potentially represents two *Image Schemas*, SCALE (parameterized to encapsulate a big quantity) and BLOCKAGE. However a speaker might produce a single gesture (i.e., overlapping the pronunciation of both words) corresponding to the meaning that is being emphasized in the context of the conversation.

Prosodic and linguistic features of the speech seem to have the potential to be the contextual markers that could be correlated with the *Image Schema* selection process (Wagner et al., 2014). Several works showed that gesture and speech timings seem to be close to each other but not exactly simultaneous. Results from Leonard and Cummins (2011) or Loehr (2012) acknowledge the correlation between gesture phases and prosodic markers while accepting slight variations. In the particular case of beat gestures, which are not constrained by meaning, the peak of the stroke seemed to be closer to the pitch emphasis (Terken, 1991). For representational gestures, it would seem that the gesture anticipates the prosodic markers of the discourse. In Kendon (1980), Kendon states that the stroke of a gesture precedes or ends at, but does not follow, the phonological peak of the utterance. In her work, Calbris also demonstrated that when constructing thoughts in a discourse, gestures tend to slightly anticipate the speech (Calbris, 2011).

Additionally, an utterance can be decomposed into a theme, the topic being discussed, and a rheme, the new information on the theme that is being conveyed (Halliday et al., 2014). Calbris observed that while enunciating the rheme of an utterance, more representational gestures are produced than in the theme (where more beat and incomplete gestures are produced) (Calbris, 2011). In other words, people tend to produce more representational gestures for accompanying and describing the new information brought by the rheme, and would align the peak of the gestures so it falls closely in time with the accentuation of the pronunciation.

## 3. Producing computationally communicative gestures

Different approaches have been investigated to address the challenge of automating gesture production and more precisely communicative gestures. Much of the existing work proposes independent reasoning units that dissociate gesture production from speech production. For instance, in Thiebaux et al. (2008), the authors developed a Behavior Realizer (Vilhjálmsson et al., 2007) capable of using different kinds of animations (computed in real-time or using pre-configured handcrafted or motion captured animations) to perform a set of requested signals. Their architecture is structured into different components communicating through a messaging system, allowing for a dynamic and responsive system and they introduced hierarchical rules to blend lower bodily functions (like posture) with higher level ones (like gaze). In the following, we present other studies that tried to do gesture alignment with the prosody or direct mapping from the surface text of the agent's discourse to gestures.

In Levine et al. (2009), the authors develop a real time system that produces gestures using prosody as input and Hidden Markov Models as the probabilistic gesture model. This model was not capable of properly handling the alignment between prosodic cues and gesture segments so in Levine et al. (2010), the authors proposed an improved version of the model using Conditional Random Fields. The result is interesting as their system produces well-aligned gestures but their meaning (and therefore the gesture shape) is not correlated with the content of the speech.

In an effort to produce gestures that were both well-aligned as well as correlated with the speech content, Chiu and Marsella integrated several data-driven, machine learning approaches^1^ to acquire a model that took lexical, syntactic and prosodic features as input (Chiu and Marsella, 2014; Chiu et al., 2015). While the approach was capable of producing well-aligned gestures correlated with the content, the results also illustrated that using machine learning to realize automatic gesture production capable of the richness of human gesture production would require a far more extensive data collection effort.

Lee and Marsella (2006) compare two approaches to generate nonverbal behaviors. The first approach, called the *literature based* approach, involves using the literature on nonverbal behavior as well as manual analysis of videos of human-human interaction to hand craft rules that map between the content of human speech and gestures. The overall design effort and complexity of such rule-based systems is very high. The second approach, a *machine learning* approach, uses a data-driven automated processes to find features (in the AMI meeting corpus Carletta et al., 2006) that are strongly associated with particular behaviors. Then, one can use those features to train models that will predict the occurrences of the behavior. The authors compare several different learning techniques (Hidden Markov Models, Conditional Random Fields, Latent-Dynamic Conditional Random Field) on syntactic features, dialogue acts and paralinguistic features, to predict speaker's head nods and eyebrow movements. The same authors used a machine learning approach in Lee and Marsella (2009) to automatically produce head movements on each part of the speech according to the dialog acts and the affective state of the agent.

Sargin et al. (2008) developed a two-level Hidden Markov Model for prosody driven head-gesture animation where the first level performs temporal clustering while the second layer does the joint modeling of prosody-gesture patterns.

In Busso et al. (2005), the authors synthesize rigid head motion from prosodic features, they also perform canonical correlation analysis to ascertain the relationship between head motions and acoustic prosodic features. The results suggested that head motions produced by people during normal speech are very different from the motions produced with an emotional state.

While prosodic information has been shown to be relevant to identify the timing and the intensity of gestures, making it a powerful input for generating beat gestures with no particular meaning or connection to the verbal content, producing representational gestures (deictic, iconic or metaphoric) requires an understanding of the information that the speaker wants to convey. Researchers aiming at producing automatically representational gestures synchronized with speech have looked at the potential of using the surface text of speech to link it with gestural representation that can convey similar or complementary information.

In Bergmann and Kopp (2009), the authors learned from an annotated corpus of spatial descriptions a Bayesian model used to predict the shape of a speaker's iconic gestures to describe the shapes of objects situated in a virtual environment (like a church). The shape of the iconic gestures is automatically computed from a geometric description of the objects in the environment. Such an approach was also used in Kopp et al. (2007) where the authors established *Image Descriptive Features* IDF (conceptually close to *Image Schemas* but used to describe geometrical and spatial features of concrete entities) and how they relate to gesture features. In both works (Kopp et al., 2007; Bergmann and Kopp, 2009), their context was a direction-giving task. They analyzed a corpus of interaction between person giving directions and exposed evidences of correspondence between the gesture features and the spatial features of the object being described. While both system allow combining multiple IDFs or geometrical description to form one gesture, which is the approach we are considering, they do not take into account the transfer of gesture properties throughout the utterance of the agent.

In Kipp et al. (2007), the authors detail their data-driven approach to build a system able to automatically generate gestures synchronized with speech. Their approach relies on the annotation of a corpus of videos of a speaker, identifying her gestures and the words associated with them, which is then used to learn the probabilities to observe particular gestures with particular words (reduced to semantic tags). Their system is capable of handling the co-articulation of gestures. When generating and selecting gestures, proximity among gestures (in terms of timing) is used to group them into gesture phrases. This grouping allows for the adaptation of the different phase existences and timings to co-articulate gestures within the same phrase and is realized thanks to a set of rules and constraints.

In most of the reviewed works, the proposed systems either tackle one aspect of our challenges (the alignment or the semantic depiction) or do not consider an intermediate representation that would allow them to reason on agent's mental state and to extend the gesture production with additional communicative intentions (such as the expression of emotion). The work that is the closest to our approach is the work conducted by Marsella and his colleagues to develop the Cerebella system (Marsella et al., 2013; Lhommet et al., 2015). In Cerebella, the studies of Lhommet (Lhommet and Marsella, 2014, 2016) and Xu (Xu et al., 2014) were combined into a complete system that extracts a mental representation from the communicative intentions of the agent to produce corresponding gestures.

In Lhommet and Marsella (2016), the authors proposed a model that maps the communicative intentions of an agent to primary metaphors in order to build a mental state of *Image Schemas*. This mental state is used to produce corresponding gestures in a second stage. In Xu et al. (2014), the authors propose a system that produces sequences of gestures that respect the notion of Ideational Units. Their system accepts as input communicative functions organized within Ideational Units (using an augmented version of the Functional Markup Language Heylen et al., 2008). This information is used to generate, using a set of defined constraints and rules, gestures that share some properties (ex. shape of the hand or location) or are co-articulated when belonging to the same Ideational Unit. However, in this work, the authors limited themselves to a restricted subset of *Image Schemas* and therefore have a limited potential for generalization.

In our work, we aim at proposing an architecture for automatically computing communicative gestures inspired by the different aspects of the challenges that have been investigated by previous researchers. Our model takes into account the linguistic structure, the prosodic information and a representation of the meaning conveyed by the agent's speech to derive gesture characteristics that are combined into coherent gesture phrases thanks to an Ideational Unit mechanism. Our model is geared to integrate a richer representation of *Image Schemas* and to be integrated in an agent system that computes in real-time the multimodal behaviors linked to additional communicative functions (such as showing emotional states and attitudes).

## 4. Image schema based gesture generator

If we were trying to replicate cognitive models proposed in the literature (e.g., Barsalou, 2009), We would need to represent mental states, and additional components such as the agent's perception, to build its inner reasoning pattern (Wilson and Golonka, 2013). As a first step, we prefer to adopt a simplified approach where *Image Schemas* are immediately tied to the speech and the gestures. We make this assumption for following reasons.

First, the focus of our investigation is on the meaning conveyed by both the verbal and the nonverbal channels. Therefore, we particularly stress the importance of the mental imagery we chose to fulfill this task. We do not reject the idea that a more faithful model would need to integrate additional reasoning components such as a grounding mechanism like in Lhommet and Marsella (2016). But our efforts focus on identifying if using a shared language between speech and gestures allows for generating more consistent multi-modal behaviors.

Second, we would have to perform more investigation on how to replicate embodied cognition mechanisms within the virtual environment of the ECA. Embodied cognition is related to the physicality of our experiences and a virtual agent does not physically experience its environment (even if it could be simulated). This is a very interesting line of research but its perspectives are outside the scope of our objectives.

**Figure 1:**
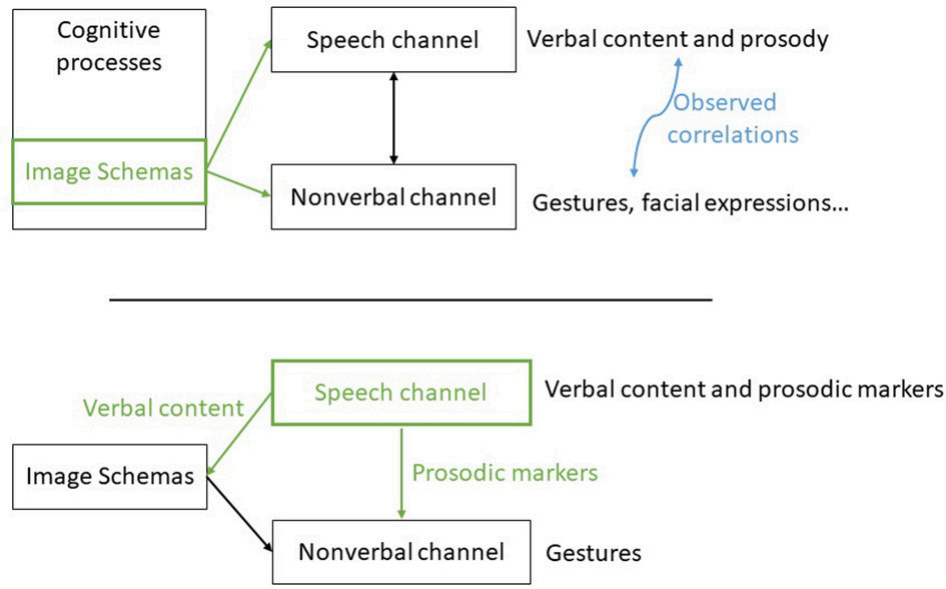
**(Top)** simplified theoretical model according to (Kendon, 1980; McNeill, 1985; Johnson, 1987), Image Schemas are used within the cognitive processes as inputs for both channels. **(Bottom)** Our framework architecture, the Image Schemas are retrieved from the text and combined with prosodic markers to generate gestures. Reproduced with the permission of the copyright holder IFAAMAS.

The model we propose is organized around the concept of *Image Schemas* as the intermediate language between the verbal and nonverbal channels. We propose an adaptation of the theoretical framework shown in Figure 1. In order to be compatible with existing speech production system, our system takes as input the speech of the agent with the prosodic markers, infers possible *Image Schemas* underlying the speech and generates the corresponding gestures. In the future, we might have a speech production system that works with *Image Schemas* and therefore which is capable of giving these *Image Schemas* to the gesture production component. But for now, we have to find a way to extract them from the text. Our architecture is composed of three levels: an *Image Schema* extractor, a gesture modeler and a behavior realizer supporting *Ideational Units*. This architecture is shown **Figure 3**.

### 4.1. Image schema extractor

The *Image Schemas* extraction component has the task of identifying the *Image Schemas* from the surface text of the agent's speech and to align them properly with the spoken utterance (for future gesture alignment). However, there does not exist a definitive list of *Image Schemas* and different researchers have proposed complementary or alternative ones. Therefore, we propose our own list adapted from the original list of Johnson (1987) and Clausner and Croft (1999). Following the idea of a parameterization of *Image Schemas* (each *Image Schema* could have different values), we decompose the SCALE *Image Schema* into smaller ordered units that would be more easily exploitable at a computer level (SMALL, BIG, GROWING, REDUCING) resulting in the following list: UP, DOWN, FRONT, BACK, LEFT, RIGHT, NEAR, FAR, INTERVAL, BIG, SMALL, GROWING, REDUCING, CONTAINER, IN, OUT, SURFACE, FULL, EMPTY, ENABLEMENT, ATTRACTION, SPLIT, WHOLE, LINK, OBJECT. This list allows us to manipulate spatial, temporal and compositional concepts (container vs. object and whole vs. split for instance). This list is not exhaustive and should definitely evolve in the future. This does not only mean adding new *Image Schemas*, but also enriching their representation. It should be possible later to parameterize *Image Schemas*, so that the gestures can be parameterized as well, and to combine them together. For instance, it should be possible to connect *Image Schemas* together to describe the evolution of an entity being discussed, like a CONTAINER being FULL or an OBJECT being an ATTRACTION or part of a SPLIT. For now, we are adopting a simplification where we are only looking to find an unparameterized *Image Schema* to match it with a gesture invariant. Gesture invariant corresponds to a feature of a gesture that is always present to carry a given meaning Calbris (2011). Our assumption is that as a first step, producing the invariant should result in a coherent animation in terms of meaning.

### 4.2. Gesture modeler

After obtaining a list of aligned *Image Schemas* for a sequence of spoken text, the gesture modeler builds the corresponding gestures.

The first step is to retrieve the gesture invariants to build the final gestures. According to the literature, the typical features of a gesture are: hand shape, orientation, movement and position in gesture space (Bressem, 2013). In Kopp et al. (2007), the authors proposed to represent gestures using the first three features augmented with a movement information on each of them. In our work, for each *Image Schemas* we want to find which features are needed to express its meaning and how it is expressed. For this task, we propose a dictionary that maps each *Image Schema* to its corresponding invariants (the features that need not to be altered to properly express the meaning). This dictionary is depicted in Table 1. This dictionary was conceived after a review of work on gesture meaning (Kendon, 2004; Calbris, 2011) and contains the minimal features required to express a specific *Image Schema*. It is not fixed and can be expanded.

**Table 1:** Association between image schemas and invariant gesture characteristics.

**Image schemas**	**Handshape**	**Position**	**Orientation**	**Movement**
UP		Up		
DOWN		Down		
FRONT		Front		
BACK		Close	Back	
LEFT		Left		
RIGHT		Right		
NEAR		Close Center		
FAR		Away	Frontward/ Downward	
INTERVAL	Flat		Inward	
BIG	Open	Away	Inward	
SMALL	Mid-closed	Close center	Inward	
GROWING				From SMALL to BIG
REDUCING				From BIG to SMALL
CONTAINER	Bowl-shape		Inward	
IN	Picking-shape		Downward	
OUT	Open spread		Outward	
SURFACE	Flat		Downward	Horizontal wipe
FULL	Closed fist			
EMPTY	Open spread			
ENABLEMENT	Open			Frontward
ATTRACTION	Closed fist			Backward
SPLIT	Flat		Inward	Abrupt downward
WHOLE	Open		Inward	
LINK	Hold			Translation
OBJECT	Conduit shape			

Once the invariants are retrieved, a gesture is built using two default gesture phases (a beginning and an end) parameterized to reflect the specific invariants. Since we are using a default template for the phases, most of the motion is predetermined but the use of the specific invariants alters significantly the shape of the gesture to express the desired meaning. For instance, if a gesture should encapsulate the *Image Schema* UP, a gesture will be built with its second phase (the stroke) that goes through a high position. In order to decide what a high position is, we follow McNeill's gesture space that divides the space used by the hand while gesticulating into 18 subspaces (upper position, lower position, periphery, center etc.) (McNeill, 1992).

### 4.3. Behavior realizer using ideational units

The final layer of our framework has the role of combining the composed gesture obtained through the previous components to produce the final animation of the virtual agent.

We define a system that follows the *Ideational Unit* model proposed by Calbris (2011) and the computational model of Xu et al. (2014). The system operates the following main functions: (1) co-articulates gestures within an *Ideational Unit* by computing either a hold or an intermediate relaxed pose between successive gestures (instead of returning to a rest pose), (2) transfers properties of the main gesture onto the variant properties of the other gestures of the same *Ideational Unit*, (3) ensures that a meaning expressed through an invariant is carried on the same hand throughout an *Ideational Unit* and (4) finally dynamically raises the speed and amplitude of repeated gestures. More precisely, to compute the relax pose of a gesture, our algorithm lowers the wrist position in 3D space; it also modifies the hand shape by using the relax position of the fingers rather than straight or closed positions. A gesture phase is held within an *Ideational Unit* when the time between the end of the gesture stroke and the beginning of the next gesture stroke is below a given threshold. To transfer properties of one gesture (here the main gesture) to the other ones, we configure their features to be identical to the main gesture, unless they were indicated as invariant. To mark the repetition of a gesture, we extend the position of the wrist in 3D space for each gesture stroke position to increase the amplitude of the gesture. We do not modify the timing of the gesture phases but since the position of the arms have been extended and their duration is the same, the speed is increased as a consequence.

This mechanism needs to know which is the main gesture of an *Ideational Unit* and what are the invariants of the gestures (in order to know which features from the main gesture can be copied to which features of the other gestures). This information is found within our dictionary of invariants. We are not working on the automatic detection of *Ideational Unit* in the text however, since this information is needed, we proposed a simplification of the approach that considers for now that a sentence is equivalent to an *Ideational Unit*. Of course, an *Ideational Unit* can span over multiple sentences or multiple *Ideational Units* could be found in a sentence, but this approximation allows us to start to manipulate this concept. In order to select the main gesture, we follow this simple rule inspired by Calbris'observations on the importance of the rheme in a sentence Calbris (2011): we choose as the main gesture the first gesture, in the sentence, built from a stressed *Image Schema* (using the prosodic markers).

### 4.4. First implementation : metaphoric gesture generation

In order to assess the relevance of our approach, we implemented a preliminary version of the system that focuses on the production of metaphoric gestures found in political speeches. We decided to explore political speeches since they are known to be richer in conceptual metaphors (Lakoff and Johnson, 1980), which in turn might lead to more metaphoric gestures (Cienki, 1998) that, according to our assumptions, should convey *Image Schemas*.

**Figure 2:**
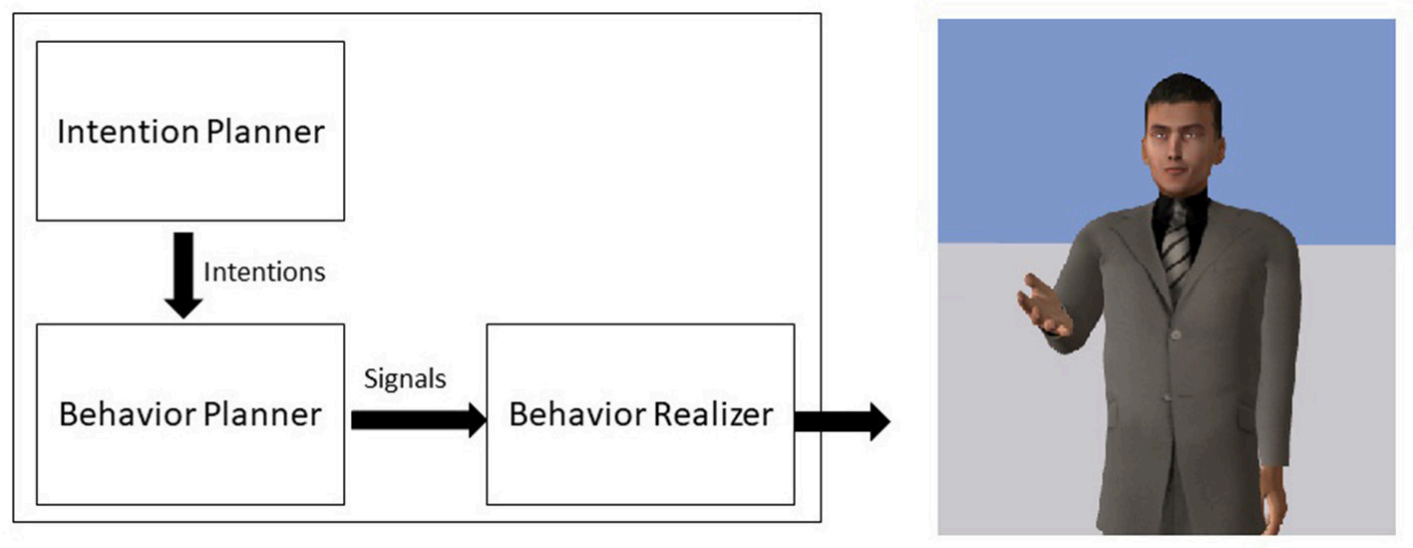
The classic agent architecture SAIBA. The Intention Planner produces the intentions of the agent, the Behavior Planner selects the appropriate signals and the Behavior Realizer computes the final animation.

**Figure 3:**
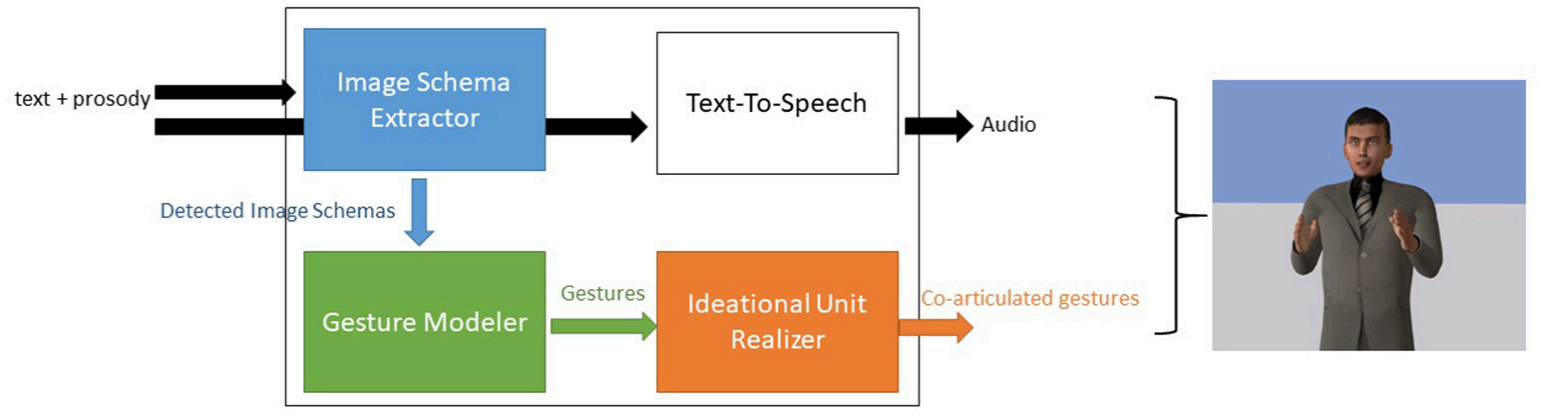
The steps and components of our architecture. The Image Schema extractor identifies *Image Schemas* from the text, the gesture modeler builds gestures using the invariants associated to each *Image Schema* and the *Ideational Unit* compatible behavior realizer produces the final sequence of gestures aligned with the audio.

We implemented the model within the agent platform Greta (Pecune et al., 2014). Greta is an agent platform, compliant with the SAIBA standard, that allows the development of components that integrate seamlessly. The SAIBA standard defines the base components of an agent which includes an Intention Planner, in charge of computing the communicative intentions of the agent, a Behavior Planner, in charge of selecting the different signals (verbal and nonverbal) to perform the intentions and a Behavior Realizer that produces the final animations (see Figure 2). In our case, we developed the *Image Schema* Extractor and the Gesture Modeler as an alternative to the Intention and Behavior Planners and we extended the Behavior Realizer in order to take into account *Ideational Units* in the production of the animations (see Figure 3). The system reads an XML-based text file (a Behavior Markup Language BML document as described in Vilhjálmsson et al., 2007) that describes the textual speech of the agent marked with prosodic and *Ideational Unit* information and produces the complete animation with the audio using a Text-To-Speech component.

#### 4.4.1. Image schemas extractor

For this first implementation of the *Image Schema* extractor, we are using an expert approach using the WordNet dictionary (Miller, 1995). In WordNet, words are organized in synonym sets. A synonym set represents a meaning, with all the words belonging to a synonym set sharing the same meaning. Each set is connected to other sets by semantic relations, giving additional information on a particular meaning. Following the hypernymic relations of a synonym set, one can obtain a synonym set with a more general meaning (for instance a hypernym of *table* is *furniture*). This organization is similar to a class inheritance system.

It is important to mention that a word might belong to different synonym sets if it can have multiple meanings. For instance, the word *table* can mean a piece of furniture or a set of data arranged in rows and columns.

Our algorithm works as follows (see Algorithm 1): for each word in the text, we use the Lesk method to disambiguate the meaning of the word and find the most likely synonym set for it using WordNet (Lesk, 1986). The Lesk algorithm compares the set of neighbors of the word being analyzed, in the current sentence, with its different definitions and chooses the definition (the synonym set) that has the most words in common with the neighbors. Then, we follow the hypernym path up in the hierarchy until we find a synonym set corresponding to our *Image Schemas* (if none is found, no *Image Schema* is returned). Using the literature on conceptual metaphors and by observing political videos, we empirically established this repertoire of synonym sets corresponding to *Image Schemas*. Several synonym sets are associated to each *Image Schema* to cover possible variations in meaning.

**Algorithm 1:** ImageSchema extraction using WordNet.

**for all** word **do**
ImageSchema = none;
SynonymSet = Lesk(word);
**while** TopNotReached() & ImageSchema == none **do**
ImageSchema = getImageSchema(SynonymSet);
MoveUpFollowingHypernym();
**end while**
**end for**

#### 4.4.2. Syntactic and prosodic selection

Instead of keeping all *Image Schemas* that were detected for every word, we select some of them by following observations from the literature in order to avoid exaggerating the gesticulations of the agent. We use OpenNLP chunker (Morton et al., 2005) to group words into phrases (e.g., noun phrases and verb phrases) and we tag one *Image Schema* per group as the main *Image Schema* of this group. We use the Stanford POS Tagger (Toutanova et al., 2003) to retrieve the syntactic role of each word and we prioritize the *Image Schemas* obtained from modifiers such as adverbs and adjectives (Calbris, 2011) as main ones unless a stressed accent is put on a particular word, in which case we prioritize the *Image Schema* coming from this word. This also leads to the selection of the main gesture of an *Ideational Unit* as seen in section 4.3. In case of multiple candidates, we randomly select the *Image Schema* for the group from them. As we saw earlier in section 2, gestures can also slightly anticipate speech (Wagner et al., 2014). In order to properly align them, we use the prosodic information to ensure that gesture strokes end at or before (up to 200 ms) pitch accents (Kendon, 2004). In Wang and Neff (2013), the authors identified through an experiment that an agent's gestures might not need to be tightly synchronized, little variations are acceptable, but should they arise, gestures should be moved earlier and not later (which is comparable to what has been found in the literature). The result is a list of *Image Schemas*, each one specifying exactly when it starts and ends in the spoken text using time markers. The prosodic information needs to be given to our system. We developed a pipeline to transform videos with subtitles into our BML format that describes the speech content (as text) along with its pitch contour. We are using OpenSmile (Eyben et al., 2013) to extract the pitch contour and *gentle*^2^ speech alignment tool to align the words with it. From there we can automatically build the BML files, that include the prosodic information associated with the text content of the speech, ready to be given to our system to generate a corpus of examples.

#### 4.4.3. Illustration

**Figure 4:**
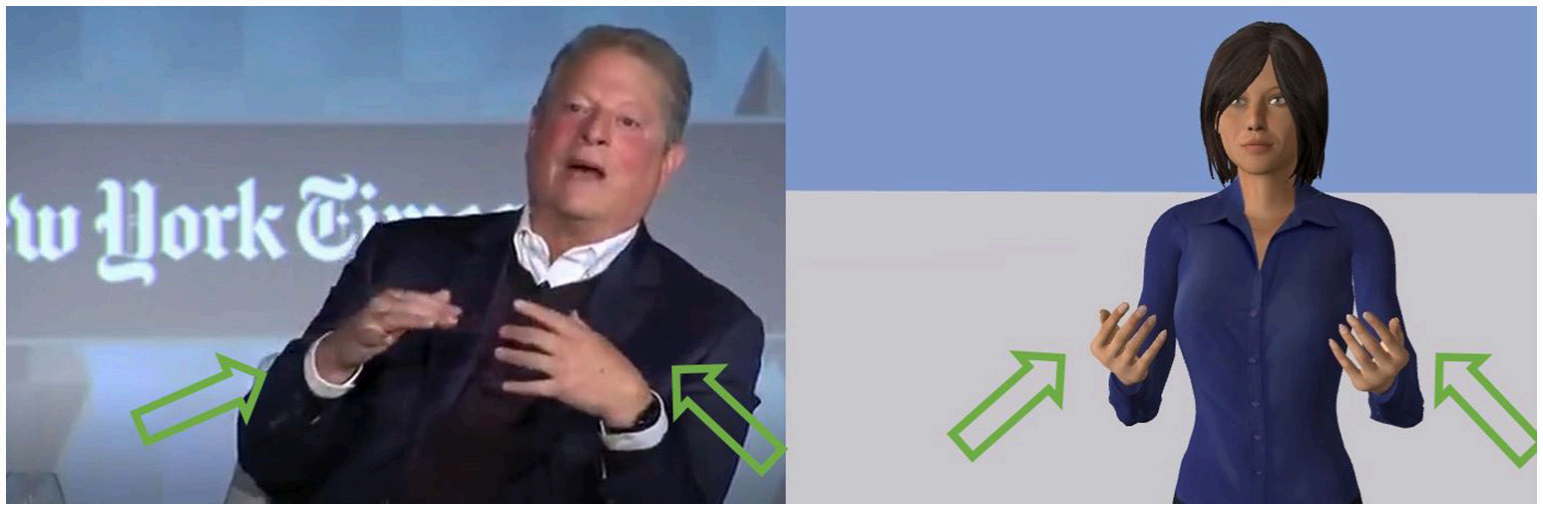
The case of the sentence “We have to get back to harvesting the wisdom of crowds.” **(Left)** The politician gather the space around him toward him. **(Right)** The agent produces a similar gesture by pulling something toward her. Reproduced with the permission of the copyright holder IFAAMAS.

To illustrate our gesture generator model, we selected a video^3^ showing a politician (Al Gore) displaying metaphoric gestures; we transcribed the textual speech and the prosodic information from the videos and let our system produce the corresponding gestures^4^. In this video, Al Gore is producing many metaphoric gestures. This video offers an interesting comparative basis to see if our model can capture the invariants of these metaphoric gestures. The output of our gesture generator model showed similarity with the input video. For each metaphoric gesture of the video, our model produced a gesture with similar timings. Some of them were carrying similar meaning as well; for the sentence “the internet is full of junk,” both the politician and our system produced a circling gesture depicting the fullness underlined in this sentence. In another example, at the beginning, the politician says “we have to get back to harvesting the wisdom of crowds” while moving his arms in a circle like he is gathering the wisdom (see Figure 4). Our algorithm captured the *Image Schema* ATTRACTION in the word *harvesting* and therefore, produced a gesture where the agent is pulling something toward her (very similar to the politician gesture).

**Figure 5:**
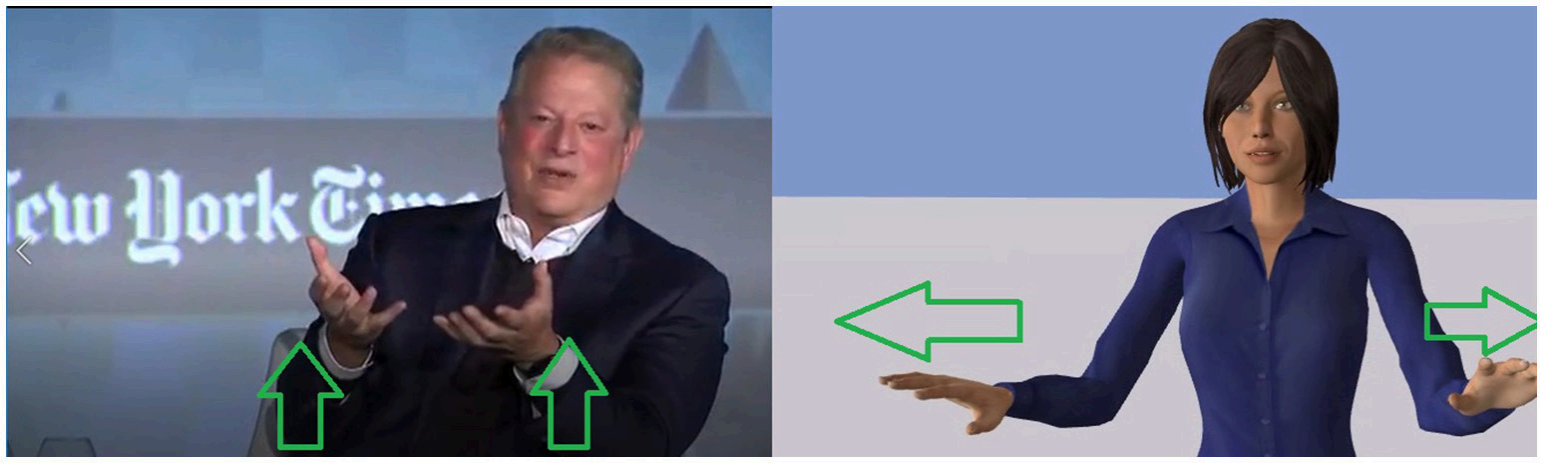
The case of the sentence “good ideas rise to the surface.” **(Left)** The politician illustrates his speech with a rising gesture, communicating a particular intention. **(Right)** The agent choses to illustrate the surface concept and thus displays an horizontal wipe gesture which illustrate a different communicative intention. Reproduced with the permission of the copyright holder IFAAMAS.

Another interesting example happened when the politician said “good ideas rise to the surface.” In the video, the politician does a gesture mimicking something going up, to accompany the verb “rise.” In our output, the *Image Schema* SURFACE, extracted from the word “surface,” was identified as the main *Image Schema* of the group rather than the UP one (that was extracted for the “rise” word). This choice resulted in the agent doing a gesture with a horizontal wipe (see Figure 5). This example is interesting as, despite being different in meaning (compared to the politician original gesture), the gesture produced by our system was still coherent with the words of the speech. In the original video, the temporal relationship between the speech and gestures varies, with gestures being perfectly in sync and others being a little bit ahead of the speech, consistent with the literature on the timing of gestures. Our system did not produce that much variability in the temporal relationship between speech and gesture, resulting in gestures having closer temporal relationship with speech in our output than in the original video. Understanding what causes this temporal variability in human communication in order to model it is another challenge that could be addressed in future work.

We observed that the output of our system did not systematically reproduce the exact gestures seen in the source video as it may select other *Image Schemas* to be highlighted with a gesture (linked to another intention the speaker wants to convey); but, nevertheless, it was able to generate animated sequences that are coherent in terms of speech-gesture mapping and synchronization. We gave as input to our gesture generator model Al Gore's speech defined in term of words and prosody. Such input does not capture all the speaker's intentions. Differences between Al Gore's gestures and output from our algorithm could arise from this lack of information. Our algorithm uses only words and acoustic information to select which metaphoric gesture to display. It does not catch which intention prevails in selecting a gesture. In the example “good ideas rise to the surface” Al Gore does a UP gesture emphasizing the emergence of good ideas while our model computed a SURFACE gesture as specified in the text.

In a traditional SAIBA architecture, we start from the intention of the agent from which we derive the signals to produce. In our system, we assume that the speech is given to us, without describing exactly what was the original intention that led to this speech. This information would be useful in order to disambiguate the meanings and to identify which word should be stressed and illustrated with a gesture. Arguably, looking to retrieve the *Image Schema* is a first step toward a mechanism that could retrieve the communicative intentions of a speaker but this is out of the scope of the current work.

## 5. Conclusion

Throughout this article, we established the foundations for developing systems capable of generating metaphoric gestures automatically from speech. We identified the key challenges for the completion of this objective, from the synchronization of speech and gestures in terms of rhythm and intensity, to a proper meaning representation and the conveying of that meaning. We discussed some of the fundamental issues raised in the psychological and embodied cognition literature on how people build and use structured representations to produce both verbal and nonverbal behaviors. From there, we proposed to use an intermediate representation between the text and the gestures inspired by *Image Schemas* that could help us solve the technical challenges of computing automatically the communicative gestures. Our approach relies on inferring automatically from the surface text of the agent the possible underlying *Image Schemas* and to combine those with the prosodic information in order to select the particular gesture characteristics to convey the imagery. In order to propose a coherent and flexible system, this process is integrated with an ideational unit compatible engine that takes care of invariant priority and co-articulation between the gestures. Our approach leverages previous studies that tackled various parts of our objectives by extending some of their functionalities and by combining them into one complete system with regards to the existing agent's standards (the SAIBA architecture). These parts include how to synchronize gestures and speech based on prosodic information, how to configure the characteristics of the gestures (hand shape, movement, orientation) to convey the desired representational meaning and finally how to combine and co-articulate these gestures into a coherent and meaningful unit of behaviors. We implemented a first version of the system in order to evaluate the potential of our approach. Our method does not always produce the same gestures as in an original video. From a technological perspective, these differences mainly come from the selection of the “important” *Image Schema* and with the speech alignment. A potential improvement for this approach would be to use Sequential Learning approach as they have proven to be an effective method to identify particular structures in text like Named Entities (Nadeau and Sekine, 2007). Additionally, we consider an utterance and its prosody profile, but we do not take into consideration other contextual factors such as what has already been said or if there are contrastive elements in the utterance. Another explanation for the differences we obtained could be that our system has a limited set of gesture invariants and, despite being able to produce coherent gestures, it cannot capture the variations or style of a speaker. An interesting alternative could be to build a stochastic model of invariants learned from a corpus of gesture data for a given speaker. This could introduce more variability and allow the reproduction of a “speaker style” like in Durupinar et al. (2016).

Alternatively, these differences might be due to some other limitations from the theoretical background we are relying on. The idea of a common mental structure is quite developed through the literature as seen by work such as Croft and Cruse (2004) or Cienki (2013) but the exact mechanism is still unknown. While the field of embodied cognition supports the idea that our physical interactions with the world shapes these structures (see Johnson, 1987; Wilson and Golonka, 2013), the process that could give different shapes (how big is big in the speaker's mind?) and importances to them (what does the speaker want to emphasize?) remains a complex system which is not fully understood yet. Exploring these models and theories with the use of virtual character capable of mimicking the human communication processes, which can be extended and manipulated, could help to investigate the details of these theories.

Whereas our approach will allow an agent to produce automatically metaphoric gestures, more investigation has to be done to ensure how to extend our system to handle other representational gestures (like deictic and iconic). Moreover, a challenge that will arise will be to combine the meaning conveyed by these metaphorical representations with the communicative intentions of the agent or with other nonverbal behaviors that can be used for turn regulation in the conversation. In the near future, we plan to evaluate our model through a perception study where participants will assess which *Image Schema* they perceive in the gestures of the virtual agent. Their feedback will be valuable to assess the progress of our approach toward an automatic generation of nonverbal behaviors as well as to inform the next steps of our research.

## Author contributions

BR wrote the manuscript with support and feedback from all the other authors. More specifically, CC participated in the review of work related to the use of prosodic information for gesture alignment. CP contributed to the formal definition of gestures used in the manuscript. SM provided assistance with the theoretical definitions coming from the embodied cognition domain.

### Conflict of interest statement

The authors declare that the research was conducted in the absence of any commercial or financial relationships that could be construed as a potential conflict of interest.

## Footnotes

^1^The work combined deep learning techniques with the temporal modeling capabilities of Conditional Random Fields to select which gesture to convey the meaning, while Gaussian Process Latent Variable Models were used to synthesize the gesture motion and co-articulate the gesture sequences.

^2^https://lowerquality.com/gentle/

^3^https://youtu.be/0ggic7bDNSE

^4^https://youtu.be/47QLONZS5zw
